# The beneficial effect of *Allium Cepa* bulb extract on reproduction of rats; A two-generation study on fecundity and sex hormones

**DOI:** 10.1371/journal.pone.0294999

**Published:** 2024-03-14

**Authors:** Sadia Suri, Saira Saeed Khan, Sadaf Naeem, Zeb Un Nisa, Nausheen Alam, Saba Majeed, Suresh Kumar, Rafeeq Alam Khan

**Affiliations:** 1 Department of Pharmacology, Faculty of Pharmacy and Pharmaceutical Sciences, University of Karachi, Karachi, Pakistan; 2 Faculty of Pharmacy, Ziauddin University, Karachi, Pakistan; 3 Institute of Pharmaceutical Sciences, Jinnah Sindh Medical University, Karachi, Pakistan; 4 Department of Pharmacology, Federal Urdu University, Karachi, Pakistan; 5 Department of Pathology, Dow University of Health Sciences, Karachi, Pakistan; Baotou Medical College, MONGOLIA

## Abstract

*Allium Cepa Linn*. (Onions) has extensively been used in traditional medicine, is one of the important Allium species regularly used in our daily diet, and has been the source of robust phenolic compounds. The current study is intended to evaluate the fecundity-enhancing effect of *A*. *Cepa* on the reproductive performance of two successive generations of rats; F_0_ and F_1_. *A*. *Cepa* extract was initially tested for in vitro antioxidant assay via DPPH and ROS, followed by in vivo toxicity testing. In the fecundity assessment, eighteen pairs of male and female rats (n = 36, 1:1, F_0_ generation) were divided into three groups and dosed with 75mg/kg and 150 mg/kg daily of *A*. *Cepa* extract and saline respectively, up to pre-cohabitation, cohabitation, gestation and lactation period. The reproductive performance, including body weight, live birth index, fertility index, and litter size, was assessed. Various parameters like Hematological, Hormonal (FSH, LH, Testosterone, estradiol), antioxidant markers (SOD, Glutathione peroxidase) and lipid profile of F_0_ and F_1_ generations were assessed with evaluation of histopathology of male and female organs. Ethanolic extract of *A*. *Cepa* showed the greatest antioxidant potential in DPPH and ROS methods. The continued exposure of the F_0_ and F_1_ generations to *A*. *Cepa* extract did not affect body weight, fertility index, litter size, and survival index. However, semen pH, sperm motility, sperm count, sperm viability, and semen volume were significantly improved in both generations. We have found pronounced fecundity outcomes in both genders of F_0_ and F_1_ generations with *A*. *Cepa* 150mg/kg/day extract as compared to control. Results showed that *A*. *Cepa* significantly increased (P < 0.05) hemoglobin, follicular stimulating hormone (FSH), luteinizing hormone (LH), plasma testosterone and glutathione peroxidase activities, while total lipid, LDL, and cholesterol were significantly decreased (P < 0.05) in both generations. Histology of both generations of animals reveals enhanced spermatogenesis and enhanced folliculogenesis with improved architecture. Altogether, the present results suggest that *A*. *Cepa* extract improved fecundity in both male and female rats by improving hormonal activities and oxidative stress.

## Introduction

Infertility can be defined as the failure to accomplish a pregnancy clinically after 12 or more months of regular unsafe sexual intercourse [1]. It has been estimated that around 15% of couples are infertile globally [2]. Within all infertility issues, human sterility attributed to male fertility issues reports for 45–50% [3], and female accounts for 46.6% of cases with polycystic ovarian syndrome (PCO_S_) constituting the principal cause [4]. To date, factors underlying the dysfunction of the reproductive system are incompletely understood. The use of recent drugs to improve fertility dysfunction is insignificant in achieving her pregnancy outcomes [5]. So, the future focus will be on formulating more viable treatment choices to prevent or treat reproductive system dysfunctions to improve fecundity.

For several years, locally grown plants in place of supplements have been used globally to vitalize, energize, and enhance male sexual functions. Several plants extract have now been identified for improving fecundity and other sexual functions, including *Parkia Biglobosa* [6], *Eugenia Uniflora* [7], *Montanoa Tomentosa* [8], *Bryonia Laciniosa* [9], *Terminalia Catappa* [10], *Tribulus Terrestris* [11] and *Lepidium Meyenii* [12]. These plants’ active constituents, including terpenoids, phenols, alkaloids, and saponins, may have a role in restoring fertility [13]. Polyphenols, especially flavonoids, are recognized to have estrogenic [14] or androgenic capabilities [15] that have a crucial role in maintaining fecundity. Such findings pointed out that flavonoids can be essential in recovering spermatozoa and spermatogenesis [5]. Sperm infertility may be caused by various factors among which ROS over production constitutes a major risk factor. Enhanced ROS production oxidatively spoil almost all biological molecules in cell involving DNA leading to enhanced sperm membrane damage, declined motility and disability of sperm to fertilize the oocyte [16].

*Allium Cepa Linn*. (Onion) is the well-known edible bulb of the Alliaceae family and is considered among the oldest cultivated species in the world [17, 18]. It contains various constituents [19] studied because of their different pharmacological properties [20]. *A*. *Cepa* has been a valuable medicinal plant for decades due to its robust free radical scavenging potential [21]. It contains antioxidants such as vitamins, selenium, glutathione, and flavonoids like isorhamnetin and quercetin [22, 23]. It has been documented that incorporating antioxidants in the diet protects sperm DNA against oxidative damage and improves sperm health [24]. Moreover, Ghasemzadeh *et al*., [25] reported that *A*. *Cepa* ethanolic extract administration significantly compensated the blood antioxidant level in the experimental polycystic ovary (PCO) model of Wistar female rats. Recent researchers claim that the presence of these anti-oxidants in *A*. *Cepa* may be beneficial and protective in reproductive functions [26–28].

Adeleye *et al*. [29] reported that *A*. *Cepa* has increased the serum levels of some hormones, luteinizing hormone (LH), and follicle-stimulating hormone (FSH); it has also improved sperm motility in birds. Another study elucidated the protective effect of *A*. *Cepa* juice against dexamethasone-induced oxidative stress during lactation in rats [30]. The cadmium-induced sperm toxicity and testicular oxidative stress were successfully attenuated after the administration of *A*. *Cepa* aqueous extract to rats [31]. The evidence shows that *A*. *Cepa* has a protective potential against testicular oxidative damage and reproductive dysfunction by stimulating the free radical scavenging system and decrementing lipid peroxidation.

*A*. *Cepa* extract presented its exclusive actions by augmenting fecundity power and diminishing fecundity-related problems. Assessment of Body weight and blood parameters provide information regarding the health of the rodents and reproductive effects of *A*. *Cepa* extract [32]. However, no research has been executed to explore the beneficial role of *A*. *Cepa* on reproduction in two generations. Therefore, a two-generational study was established to explore the beneficial and protective use of *A*. *Cepa* extract on fecundity and reproductive hormones in experimental animals.

## Materials and methods

### Extraction of plant material

*A*. *Cepa* was purchased from a local market in Karachi, Pakistan, and was distinguished at Herbarium and Botanic Garden, University of Karachi and has got an identification number; G.H. No. 956008 (Allium Cepa L. of Alliaceae family).

The *A*. *Cepa* bulbs were washed with distilled water. The outer layer of the bulbs was peeled off manually and rinsed with sterile distilled water, then air dried properly, cut into small pieces, and chopped. The pieces were soaked in 96% ethanol for five days (100 grams of the sample soaked in 200 ml ethanol) at room temperature (25°C to 30°C) with intermittent shaking. The ethanol extract was filtered and later evaporated to dryness at 20°C using a rotary evaporator (Buchi Rotavapor, Germany) and stored at 2°C– 8°C in a refrigerator [33].

### Chemicals

Ferric Chloride, Petroleum ether, Ethanol (96%), Formalin, Potassium hydroxide, Glacial acetic acid, Sulphuric acid (concentrated), Sodium Hydroxide, Mayer’s and Dragendoff’s reagents, HCl 1%, Cholesterol reagent, Conjugate solutions for testosterone and estradiol, Tetramethylbenzidine (TMB), Stop solution, HRP (Horseradish peroxidase) Conjugate reagent, FBS (Fetal bovine serum), PBS (Phosphate-buffered saline), Na_2_CO_3_ (30 g/L) were procured from Sigma Chemicals Company (St Louis, MO, USA). Etoposide, 0.1 mM DPPH, DMEM (Dulbecco’s Modified Eagle Medium) and Penicillin-streptomycin were procured from Sigma–Aldrich Co., USA. Gallic acid and Folin-Ciocalteu reagent were obtained from Merck (Darmstadt, Germany). Olive oil was purchased from Saeed Ghani Ltd., Pakistan. All chemicals used in this study were of analytic grades. Elisa Kits was used to assay the levels of FSH (serial number-54371), LH (serial number-52167), plasma testosterone (serial number-55378), SOD and Glutathione (Glory Science Co.2022#45).

### Preliminary phytochemical screening

The phytochemical tests of ethanolic extract, including tannins, alkaloids, saponins, flavonoids, glycosides, and phlobatannins, were performed by methods mentioned already with little modifications [34–36].

### Total phenolic content analysis

The method used for total phenolic content described previously by Lee *et al*. [37] utilizes Gallic acid as a standard to obtain a curve by plotting. With the aid of serial dilutions utilizing deionized water, different concentrations of dilutions; 50.0, 25.0, 12.5, 6.2, and 3.1 mg/ml, were formulated. Constituent volumes were added to a single vessel: reagent 100 μL: Folin-Ciocalteu; 20 μL: sample-blank (DI water)-standard gallic acid; 80 μL: Na_2_CO_3_ (30 g/L). The microplate was allowed to incubate for 15 minutes at room temperature, followed by taking an absorbance reading at 540 nm utilizing a UV-VIS spectrometer (Genesys™ 10S UV-Vis, Thermo Scientific, USA). Dilutions were executed on the extract as considered necessary for the values of absorbance to fit among the standard curve. The results were declared as mg/g of gallic acid equivalent.

### Antioxidant assays

#### Antioxidant assay of *A*. *Cepa* by DPPH

An upgraded DPPH assay (1,1-diphenyl-2-picrylhydrazyl) was selected to evaluate the tested compound. The DPPH radicals diminish at 517 nm of absorbance in the presence of antioxidant potential. The test compound *A*. *Cepa* at different concentrations (10, 20, 100, 300 μg/ml) was added to 0.1mM DPPH (3ml) and allowed to react. The mixture was swirled and incubated for 30 min at room temperature. A microplate reader (SkanIt Software 5.0 of RE, ver. 5.0.0.42) was utilized to measure the absorbance at 517 nm. The test compound reducing power was calculated with the absorbance of ascorbic acid, which served as control [38].

Radical Scavenging Activity was equated in percentage via equation.


$$\%\;RSA=\{(A_{control}-A_{sample})/A_{control}\}\times 100$$

#### Antioxidant assay of *A*. *Cepa* by ROS

Primary cortical cells were used for the determination of Reactive Oxygen Species (ROS) generation. The method [39] was adopted with some modifications. The untreated cells were used as a control. Cortical cells were harvested from the brain of two-day-old rat pups. Cells were grown in DMEM affixed with 10% FBS and then 1% penicillin-streptomycin (100 units/mL-100 μg/mL) respectively. Adhesive cell monolayers were cultured into T25 tissue culture flasks to be used for further experiments. The cortical cells were incubated at 37°C in a humidified atmosphere having 5% carbon dioxide (CO_2_). ROS Intracellular levels of living cells were assessed quantitatively by employing a fluorescent probe, 2′,7′ dichlorodihydrofluorescein diacetate (DCF-DA). Primary cortical cells were subjected to various concentrations of extract for 12 h. Untreated or treated cultures were allowed to incubate in the dark with 10 μM DCF-DA at 37°C for 45 minutes, and later on, washed with PBS. Relevant ROS level changes intracellularly were observed by fluorometric detection of DCF by using a fluorescent Microplate Readers RE, ver. 5.0.0.42 at emission and excitation wavelengths of 530 nm and 485 nm, respectively. The fluorescence intensity level of DCF is proportional to the ROS level generated intracellularly. Percentage Cell Viability can be calculated by formula [40].

#### Experimental animals

In the experimental fecundity study, we used 36 healthy animals comprising 18 males and 18 females Wistar rats. The animals were bought from the animal house, H.E.J, University of Karachi, and were kept in plastic cages having proper ventilation under standard temperature conditions (25°C to 30°C) with 12 h light and dark cycle. The animals were given pelleted rodent food. The weight of animals ranged between 170g to 190g. The animals were placed in an animal house at the Department of Pharmacology, University of Karachi.

The experimental protocol fulfills the guidelines regarding the appropriate use and care of laboratory animals [41], and the experimental protocol and design were permitted by the Board of Advanced Studies and Research (BASR), Resolution No. 10(P)07, ASRB/No./05071/Pharm., University of Karachi.

#### Animal ethical approval

The study was performed under the approval of the Board for Advanced Studies and Research dated 28-03-2019 (reference No 05071/pharm), University of Karachi and Pharmacology Departmental Research Committee, for the use of animals according to the National Institute of Health guidelines (NIH guidelines Islamabad, Pakistan) [42].

#### Brine shrimp toxicity testing

Toxicity of *A*. *Cepa* was evaluated to rule out % Mortality with the help of the Brine shrimp method (Artemia Saline). Etoposide served as a positive control [43].

#### Acute oral toxicity

The study on acute oral toxicity was executed according to the OECD-404 guidelines [44]. Both genders’ rats were used and supplied with distilled water and fed with *adlibitum*. Twenty rats were randomly divided into four groups (n = 5). A single oral dose of ethanolic extract of Allium Cepa, 300 mg/kg, 600 mg/kg, and 1200 mg/kg were administered as per body weight respectively, to each group in comparison to the control. The rats were examined for toxicity signs like behavioral changes and mortality for 48 hours [45].

#### Fecundity assessment

The study was conducted for a total duration of seven months. A modified method of Vohra *et al*. [46] was used. Eighteen pairs of rats (n = 36, F_0_ generation) were placed in separate cages.

In the fecundity study, healthy and sexually mature male rats aged 12 weeks (155.42 ± 1.89 g) and female rats aged 14 weeks (169.1 ± 2.16 g) were used. Rats were isolated and evaluated for any gross symptoms of injury or disease. After one week of acclimatization, six experimental groups were formed. Three groups were of male rats, each comprising of six rats (n = 6), while the rest of the three groups comprises of female rats only (n = 6).

In male rat groups, Group, I served as control and administered distilled water daily; Group II animals were given low dose *A*. *Cepa* extract of 75 mg/Kg/day while Group III animals were given *A*. *Cepa* extract at the high dose of 150 mg/Kg/day [47, 48]. A similar protocol was followed for the female group as well. All rats were dosed for 30 days before mating (Pre- Cohabitation) by gastric gavage between 14:00 and 16:00 h. Female rats were subjected to the soiled bedding of an adult male rat to align their estrus cycle (female rats were fertile and receptive during this period) [49]. During mating each male rats were individually paired with female rats of each group in separate cages for seven days (1:1). Inseminated females were separated from males and placed in cages after conception. The doses of *A*. *Cepa* were given continuously to female rats during cohabitation (21 days), gestation (21 days), delivery and lactation till postnatal day of 22. When pups were delivered, all F_0_ animals (male and female) were deeply anesthetized with intraperitoneal Ketamine (10 mg/Kg) and Xylazine (5 mg/Kg) [50], to alleviate the suffering of rats. Blood samples were taken from the aorta and the animals were sent to necropsy. Subsequently, reproductive organs were taken for histopathological assessment.

Fertility indices (fertility index, the number of pups, survival index) were estimated. Offspring were counted and observed for any abnormality on postnatal days 0, 4, 7 14, and 21. Body weight was recorded weekly before, during mating, and in gestational periods. All F_0_ male/female parental animals were monitored twice daily for toxicity signs.

For male rats’ fecundity assessment, the procedure described by Quadri & Yakubu [51] was selected to prepare the semen from the epididymis. The Epididymis was carefully detached and lacerated with scissors from the testis. The semen was aspirated 10 μl from the caudal epididymis of every rat by using a capillary tube and transferred into Petri dishes already having 1 ml, 0.1 M phosphate buffer having 7.4 pH. The homogeneity was maintained by slightly swirling each dish. The sperm cells were permitted to separate in the solution at 37°C for 10 min. Afterward, the semen volume, sperm count, pH, motility, and viability were estimated using standard methods.

For F_1_ generation study, pups were grown up to 4 weeks then the same doses of the *A*. *Cepa* extract were given to 4 weeks old pups (according to their bodyweight) during their growth, adulthood, and up to breeding. After one month, 8 weeks old rats were permitted mating by making pairs on a 1:1 basis within same treatment group, preventing sibling-mating, for 7 consecutive days. The vaginal plug was noticed to confirm the pregnancy of each female rat. Pregnant female rats of the F_1_ generation were separated from male rats and placed individually. *A*. *Cepa* extract continuously given during their pre-cohabitation, cohabitation period, gestation, and lactation until the F_2_ generation pups were weaned. Gestated female rats were permitted to naturally deliver pups (F_2_ generation).

Afterward, all F_1_ generation animals were anesthetized and examined as described previously. The same protocol of the F_0_ generation was followed for both F_1_ and F_2_ generation till the birth of F_2_ pups. For all F_1_ generation female rats’ fecundity assessment, the above procedure for determining fertility indices in F_0_ generation animals were used as described by Vohra *et al*. [46]. Similarly, for all F_1_ generation male rats’ fecundity assessment, the procedure described by Quadri & Yakubu [51] was selected as discussed above in F_0_ generation animals.

#### Reproductive performance of F_0_ and F_1_ rats

The reproductive parameters studied were mentioned in the form of ratios, weights, and indices that analyze all stages starting from conception till weaning [46]. These fertility parameters were computed as follows:

Fertility index (%) = (Females count giving birth/females count lived together) × 100

Litter size = count of pups/count of gestated females

Live birth index (%) = (count of pups alive at day 0/count of pups born) × 100

Survival index—4 days (%) = (count of pups alive on day 4/ day 0 alive pups count) × 100

Survival index- 21 days (%) = (count of alive pups on day 21/ day 4 alive pups count) × 100

#### Blood parameter assessment

When pups were delivered, all F_0_ and F_1_ generation male and female animals were anesthetized with Ketamine and Xylazine. Blood samples were taken from the aorta for biochemical assessment and kept in B-Ject gel clot activator vacuum tubes under aseptic conditions. Samples were centrifuged in an Eppendorf machine at 4000 rpm for 10 min. The separated serum was kept at -80° C for hormonal and biochemical testing. All samples were analyzed for hematological (Sysmex KX-21 hematological analyzer) [52], Total lipid profile (LDL, VLDL, HDL, Triglycerides, Cholesterol), hormonal estimation (FSH, LH, testosterone and estradiol [53, 54] were assayed via ELISA Roche Diagnostics, Basil). SOD and Glutathione (GPx) were estimated by the kit acquired from Glory Science Co., Ltd. (Glory Science Co.2022#45).

#### Histopathological evaluation

For histopathological evaluation, the ovaries and testis of the selected rats were removed and cleaned from adherent connective tissues, then fixed in 10% formalin. Fresh tissue samples were dehydrated by exposing ethanol and dimethyl benzene of different concentrations (twice) at room temperature. Then, sex organs were embedded with paraffin at 60° C and cut into sections. Blocks of paraffin were cut serially and longitudinally at 4 μm thickness, followed by Hematoxylin-Eosin staining. Lastly, the stained sections were dehydrated and sealed with coverslips and imaged under microscope (Bio Base XS-208, China). Stained slides were analyzed and compared in groups [55].

### Statistical analysis

Statistical analysis was implemented using the SPSS software (version 20.0). Multiple groups were compared by one-way analysis of variance applying the Bonferroni post-hoc test. The results were considered statistically significant if the value lies between P < 0.05 and highly significant when P < 0.01.

## Results

### % Yield and total phenolic content of *A*. *Cepa* bulb

*A*. *Cepa* bulb (12 Kg) was extracted with 96% ethanol and the total yield percentage of *A*. *Cepa* extract was found to be 10%.

Total Phenolic Content was determined in the extract of *A*. *Cepa* bulb by the method of Folin-Ciocalteu (F–C) using gallic acid as the standard. Absorbance values were determined at various concentrations of gallic acid to construct the calibration curve. The total phenolic content of the extract was estimated from the regression equation of the calibration curve (*Y*  =  1.012*x*; *R*^2^  =  0.996) designated as gallic acid equivalents (GAE) (63 mg/g GAE) (Table 1).

**Table 1:** Total phenolic content in allium cepa.

Sample	Total Phenolic Content (mg/g GAE)
**Allium Cepa**	**63**

#### Acute oral toxicity

Regarding the safety of *A*. *Cepa* L. plant, acute toxicity study disclosed no signs of toxicity with no mortality in rats. However, some dose-dependent behavioral changes were observed in high-dose (1200 mg/kg) treated groups, including depression, clustering, folding and sleeping, unsteady gait, and loss of appetite. The results regarding Acute toxicity (LD_50_) of *A*. *Cepa*, by using Karber Method, is mentioned below (Table 2). The arithmetic method of Karber was utilized for calculation.

**Table 2:** Determination of acute toxicity (LD_50_) in rats using karber method.

Dose	Dose Difference	No. of dead Animals	Mean Mortality	Dose Difference- Mean Mortality
**300**	**0**	**0**	**0**	**0**
**600**	**300**	**0**	**0**	**0**
**1200**	**600**	**0**	**0**	**0**
**Control**	**0**	**0**	**0**	**0**

Mean Mortality = 0, Σ (Mean Mortality × Dose Difference) = 0

n = 5.

LD50 = Highest Dose–Σ (Mean Mortality × Dose Difference)/n.

Therefore, LD50 = 1200–0 = 1200 mg/kg.

### Preliminary Phytochemical Screening

The presence of *A*. *Cepa* constituents was mentioned in Table 3. The qualitative phytochemical analysis reveals that *Allium Cepa* possesses tannins, flavonoids, terpenoids, glycosides, saponins, phenols, and phlobatannins.

**Table 3:** Phytochemical analysis of *Allium Cepa*.

Phytochemicals	*Allium Cepa*
Phlobatannins	+
Flavonoids	+
Glycosides	+
Alkaloids	-
Saponin	+
Phenols	+
Terpenoid	+
Tannin	+

The presence of phytochemicals is represented as +, while absence is denoted as–

### Antioxidant assays

#### Antioxidant Assay of *A*. *Cepa* by DPPH

The % inhibition of *A*. *Cepa* extract at different concentrations is shown in Table 4. The extract was observed with radical scavenging potential by DPPH utilizing Ascorbic Acid as a reference standard. Among various concentrations, *A*. *Cepa* exhibited the highest activity at 100 μg/ml and 300 μg/ml, i.e., 84.92 ± 0.11% and 80.27 ± 1.20%, respectively, while Ascorbic acid exhibited the strongest scavenging activity of 88.14 ± 1.28% at 300 μg/ml.

**Table 4:** % Inhibition of *Allium Cepa* at different concentrations.

Samples	Concentration	% Inhibition
	(μg/ml)	(Mean ± SD)
***Allium Cepa* Ethanolic**	**10**	**74.37 ± 2.15**
**Extract**	**20**	**76.12 ± 0.56**
	**100**	**84.92 ± 0.11**
	**300**	**80.27 ± 1.20**
**Ascorbic Acid**	**10**	**75.96 ± 0.92**
**(Standard)**	**20**	**77.94 ± 2.02**
	**100**	**81.8 ± 1.47**
	**300**	**88.14 ± 1.28**

#### Antioxidant Assay of *A*. *Cepa* by ROS

**Fig 1:**
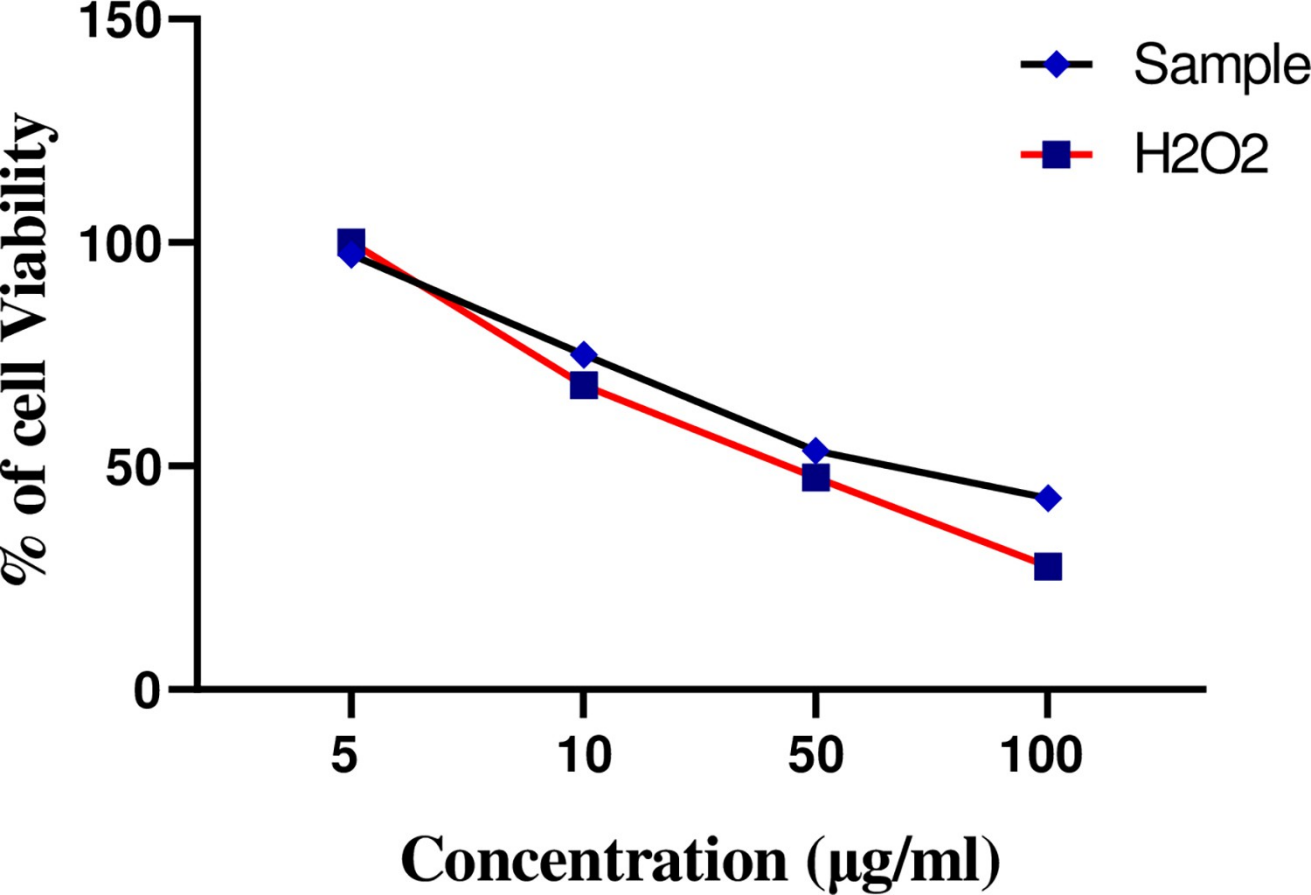
Percentage viability of allium cepa in comparison to H_2_O_2_.

The DCF-DA method was employed to determine the ROS production and % Viability of *A*. *Cepa* extract on primary cortical neuronal cells was found nontoxic and significantly prevented H_2_O_2_-induced oxidative damage. % Cell viability in neuronal cells after incubation using indicated concentrations of *A*. *Cepa* is shown in Table 5, Fig 1, which shows no cytotoxic effect in neuronal cells at concentrations up to 50 (μg/ml). However, at the dose of 100 (μg/ml), *A*. *Cepa* extract shows protective efficacy against H_2_O_2_-induced toxicity on neuronal cells.

**Table 5:** Percentage cell viability of *Allium Cepa* in comparison to H_2_O_2_.

Sample	Concentration	% Viability
	(μg/ml)	
	**5**	**97.32**
***A. Cepa* Ethanolic**	**10**	**74.92**
***B.* Extract**	**50**	**53.4**
	**100**	**42.81**
**H**_**2**_ **O**_**2**_	**5**	**100**
	**10**	**68**
	**50**	**47.49**
	**100**	**27.61**

#### Brine shrimp lethality testing

The *A*. *Cepa* extract was assessed for brine shrimp lethality studies and the results were mentioned in Table 6. No significant toxicity to brine shrimp Artemia salina was found with *A*. *Cepa* extract at different concentrations. The *A*. *Cepa* extract shows significant results, which represent that the extract is biologically safe. The % mortality of *A*. *Cepa* at 1000 μg/ml concentration is 16.66% compared to standard Etoposide, which shows 100% mortality at 1000 μg/ml, indicating the extract is significant and safe for use.

**Table 6:** Percentage mortality of Allium Cepa as compared to control and standard.

Samples	Concentration (μg/ml)	No. of Shrimps	No. of Survivors	% Mortality
Control Distilled Water	10	30	30	0
	100	30	30	0
	1000	30	30	0
Standard (Etoposide)	10	30	5	83.33
	100	30	1	96.6
	1000	30	0	100
Allium Cepa ethanolic extract	10	30	29	3.33
	100	30	26	13.33
	1000	30	25	16.66

#### Fecundity assessment

The two-generation fecundity study was conducted for seven months that showed no congenital abnormalities in any of the pups of all the groups of F_0_ and F_1_ generations. The body weights of F_0_ and F_1_ animals are mentioned in Table 7, representing insignificant differences among generations and control.

**Table 7:** Effect of A. Cepa extract on weekly body weight (g) of F_0_ and F_1_ generation female rats as compared to control.

Weeks	F_0_			F_1_		
	Control	T1 (75 mg/Kg)	T2 (150 mg/Kg)	Control	T1 (75 mg/Kg)	T2 (150 mg/Kg)
**I**	**167.5 ± 3.28**	**173.5 ± 2.43**	**167.8 ± 1.97**	**170.5 ± 2.95**	**165.33 ± 1.45**	**170.67 ± 2.04**
**II**	**169 ± 3.03**	**172.6±0.80**	**166.33±0.66**	**171.5 ± 3.14**	**164.8±0.70**	**169.6±0.66**
**III**	**171.17 ± 2.98**	**172.5±0.671**	**165.17±0.70**	**172.5 ± 3.19**	**164.6±0.88**	**168±0.73**
**IV**	**173.5 ± 3.19**	**173±0.85**	**166±0.57**	**174 ± 3.33**	**165±0.258**	**170.6±0.71**
**V**	**175.5 ± 3.04**	**174.3±0.33**	**168.3±0.91**	**176 ± 3.25**	**166.17±0.47**	**171.33±0.33**

n = 6, Mean ± SEM; F_0_ presents Parent Generation, while F_1_ presents 1^st^ Generation, T1 shows low dose group while T2 shows high dose group.

The number of pups was counted in each generation and noticed for congenital abnormalities. In all groups, no congenital abnormality was observed. The number of pups increased significantly in the first and second generations compared to the control group (df 5, 30; F 5.51; P < 0.05).

Post Hoc analysis through the Bonferroni test revealed that high-dose administration of *Allium Cepa* produced statistically significant differences in the number of pups in both generations compared to low dose group and control group rats, as shown in Table 8.

**Table 8:** Effect of A. Cepa extract on the number of pups of F_1_ and F_2_ generation as compared to control.

F_1_			F_2_			P Value
Control	T1 (75 mg/Kg)	T2 (150 mg/Kg)	Control	T1 (75 mg/Kg)	T2 (150 mg/Kg)	
**4.50 ± 0.56**	**6 ± 0.57**	**7 ± 0.51** ^*^	**4.17 ± 0.30**	**6.50 ± 0.88**	**7.50 ± 0.42** ^*^	**P < 0.05**
						**(df5,30 F 5.51/ P 0.001)**

n = 6, Mean ± SEM; *P < 0.05 significant as compared to control.

F_1_ presents 1^st^ generation Pups, while F_2_ presents 2^nd^ generation Pups, T_1_ shows low dose group while T_2_ shows high dose group.

#### Reproductive performance

The reproductive performance was assessed by observing several parameters, including live birth index, fertility index, and litter size. Insignificant changes were found in all treated groups’ live birth, fertility, and survival indexes (P > 0.05). All these parameters were improved in all treated groups, just like the control group; however, a significant increase in litter size was noticed in the treated group in both generations (P < 0.05) when compared to the control group, as shown in Table 9.

**Table 9:** Developmental findings in F_1_ and F_2_ rat pups.

Parameters	F_0_ Parents/F_1_ Pups			F_1_ Parents/F_2_ Pups			P-Value
	Control	T1 (75 mg/Kg)	T2 (150 mg/Kg)	Control	T1 (75 mg/Kg)	T2 (150 mg/Kg)	
**No. of Pregnant Rats**	**6**	**6**	**6**	**6**	**6**	**6**	**P > 0.05**
**Litter Size**	**4.5**	**6**	**6**	**4.16**	**6.5**	7.5*	**P < 0.05**
**Fertility Index (%)**	**100**	**100**	**100**	**100**	**100**	**100**	**P > 0.05**
**Live Birth Index (%)**	**100**	**100**	**97.61**	**100**	**100**	**100**	**P > 0.05**
**Survival index at day 4 (%)**	**100**	**100**	**100**	**100**	**100**	**100**	**P > 0.05**

F_0_ presents Parent Generation, while F_1_ presents 1^st^ Generation, F_2_ presents 2^nd^ Generation, T_1_ shows low dose group while T_2_ shows high dose group. n = 6. Mean ± SEM; *P < 0.05 significant as compared to control.

Additionally, in male rats, parameters like semen pH, sperm motility, sperm count, sperm viability, and semen volume are crucial indices of male fecundity as they are key markers in epididymal maturation and testicular spermatogenesis. All these parameters were significantly improved (P < 0.05) in all treated groups when compared to the control group, as shown in Table 10.

**Table 10:** Male fecundity parameters.

	F_0_ Parents/F_1_ Pups			F_1_ Parents/F_2_ Pups			
Parameters	Control	T1 (75 mg/Kg)	T2 (150 mg/Kg)	Control	T1 (75 mg/Kg)	T2 (150 mg/Kg)	P Value
**Semen pH**	**7.28** ± **0.03**	**7.32 ± 0.04**	**7.32±0.22**	**7.13±0.11**	**7.32±0.04**	**7.32±0.22**	**P > 0.05**
**Semen Volume (ml)**	**2.05**±**0.01**	**2.34** ± **0.04**	**2.51**±**0.02**	**2.05**±**0.01**	**2.48**±**0.06**	**3.1±0.10**	**P < 0.05**
Sperm Count (×10^6^ sperm/ml)	**62.0** ±**1.22**	**61.00**±**6.78**	**89.00**±8.12*	**60.00**±**1.2**	**63.02**±**6.78**	**69.00±8.1** *	**P < 0.05**
**Sperm viability (%)**	**60.0** ± **5.50**	**67.40**±**6.20**	**95.00**±6.94*	**58.0**±**5.42**	**67.34**±**6.12**	**97.30±7.2** *	**P < 0.05**
**Sperm Motility (%)**	**50.80**±**3.20**	**62.00**±**5.27**	**82.20**±6.81*	**48.1**±**4.21**	**63.00**±**5.72**	**88.22±6.9** **	**P < 0.05**

F_0_ presents Parent Generation, while F_1_ presents 1^st^ Generation, F_2_ presents 2^nd^ Generation, T_1_ shows low dose group while T_2_ shows high dose group. n = 6. Mean ± SEM

*P < 0.05 significant

** P < 0.01 highly significant as compared to control.

#### Hematological parameters

**Fig 2:**
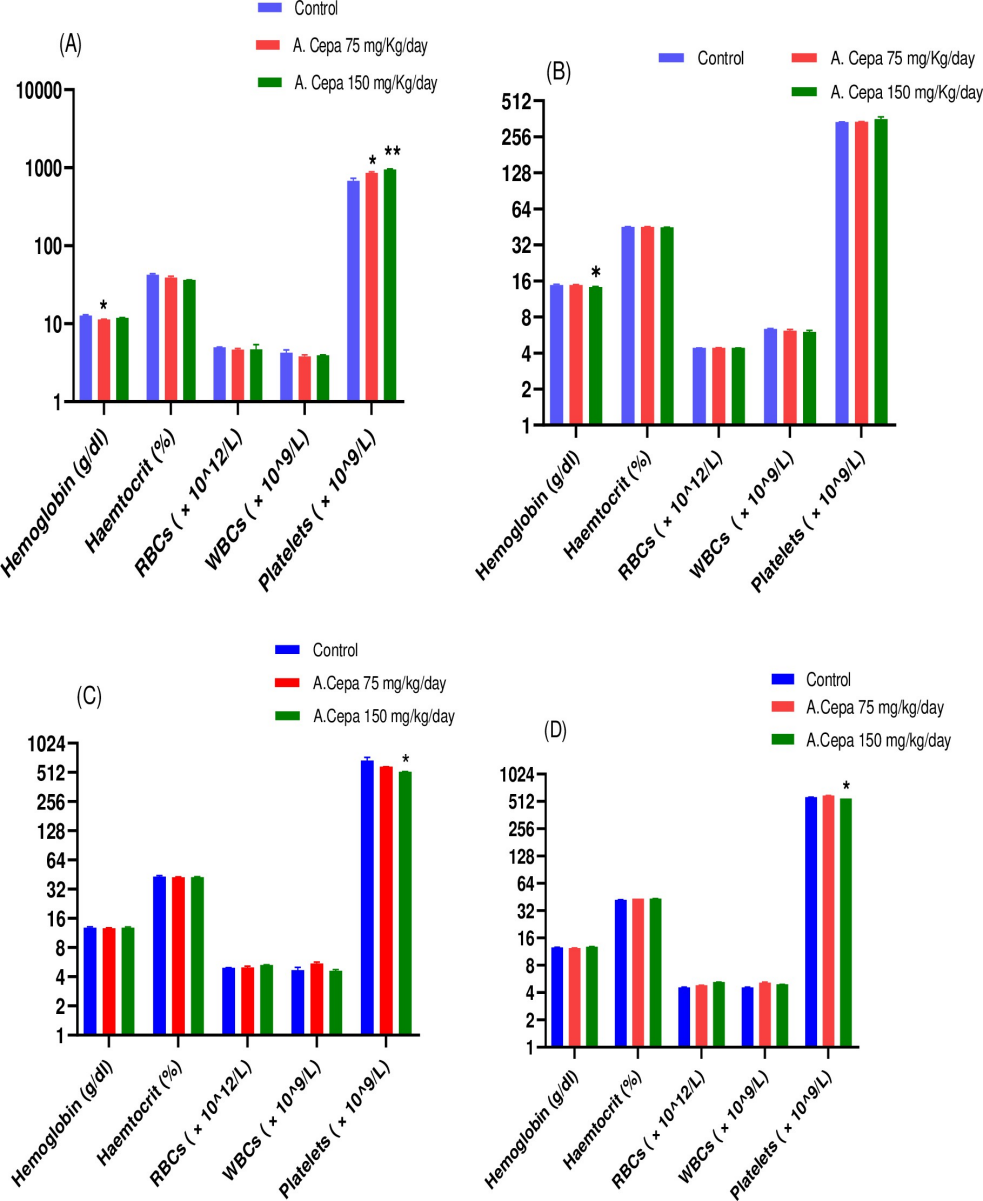
Effect of A. Cepa extract on hematological parameters. (A) F_0_ Male (B) F_0_ Female (C) F_1_ Male (D) F_1_ Female. n = 6, Mean ± SEM; *P < 0.05 significant; ** P < 0.01 highly significant as compared to control.

Hematological analysis showed the non-significant result in male and female rats, including Hb, RBCs and WBCs. However, on Platelets, highly significant results (P < 0.01) in male F_0_ generation rats (df 2,15; F 14.56; P < 0.01) were found as shown in Fig 2. Similarly, F_1_ generation male and female rats showed significant results (df 2,15; F 6.62; P < 0.05), (df 2,15; F 6.66; P < 0.05), respectively, on platelets.

#### Biochemical analysis

**Fig 3:**
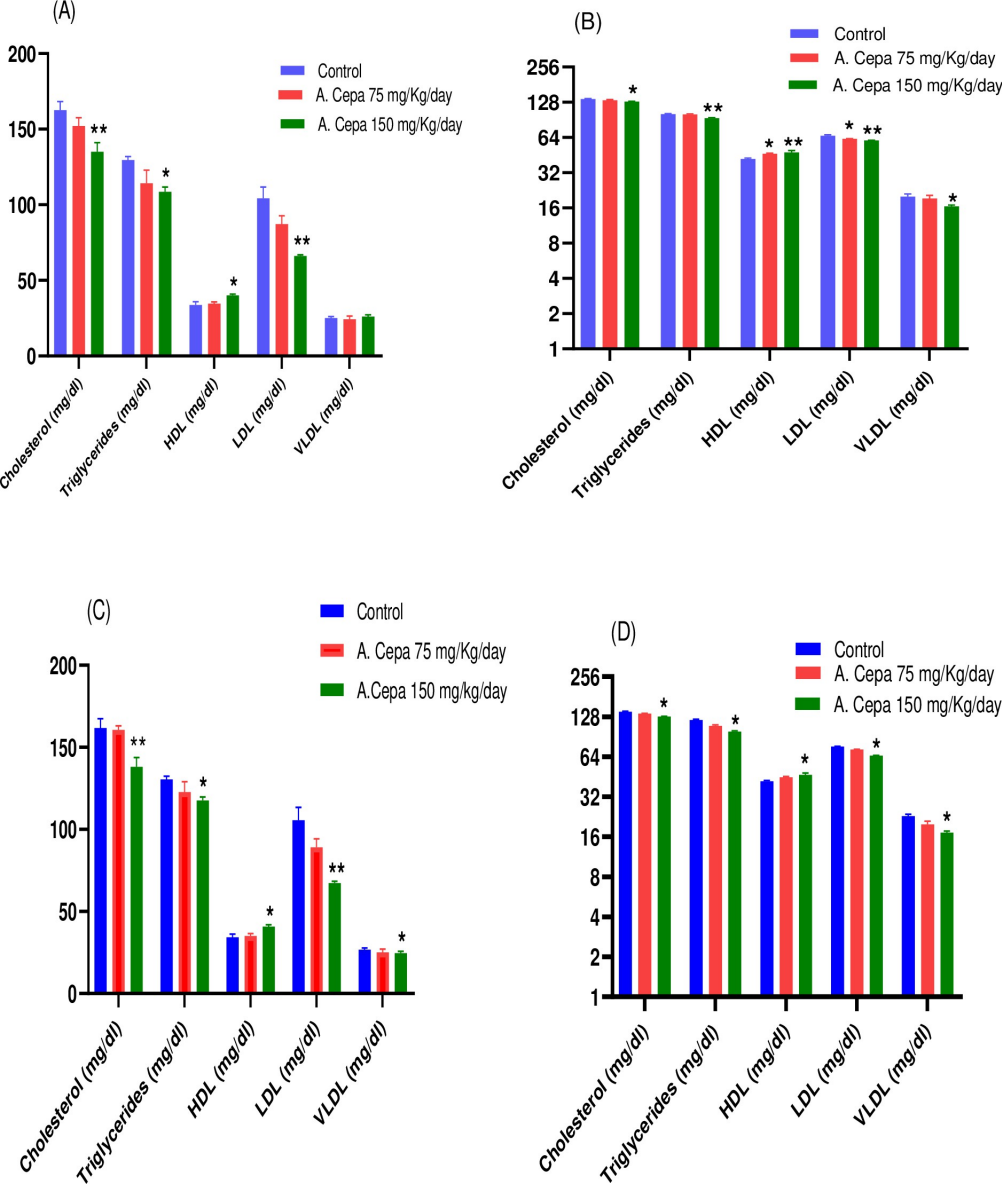
Effect of A. Cepa extract on biochemical parameters. (A) F_0_ Male (B) F_0_ Female (C) F_1_ Male (D) F_1_ Female. n = 6. Mean ± SEM; *P < 0.05 significant; **P < 0.01 highly significant as compared to control.

Biochemical Analysis showed statistically highly significant reduction in Cholesterol (df 2,15; F 5.68, 7.70; P < 0.01), LDL (df 2,15; F 12.76, 12.48; P < 0.01), significant reduction in triglycerides (df 2,15; F 3.91, 2.51; P < 0.05) and raised HDL effects (df 2,15; F 4.85, 5.42; P < 0.05) of F_0_ and F_1_ generation male animals respectively as shown in Fig 3. Correspondingly female animals of F_0_ generation showed highly significant reduction in Triglycerides (df 2,15; F 15.10; P<0.01), LDL (df 2,15; F 14.24; P<0.01) and a raised HDL (df 2,15; F 6.35; P<0.01) effect in comparison to control. Similarly, F_1_ generation female animals showed significant reduction in Cholesterol (df 2,15; F 46.66; P<0.05), Triglycerides (df 2,15; F 50.55; P<0.05), LDL (df 2,15; F 185.8; P<0.05) and VLDL (df 2,15; 10.87; P<0.05) and a raised HDL effect (df 2,15; F 4.82; P<0.05) when compared to control group.

#### Hormonal parameters

**Fig 4:**
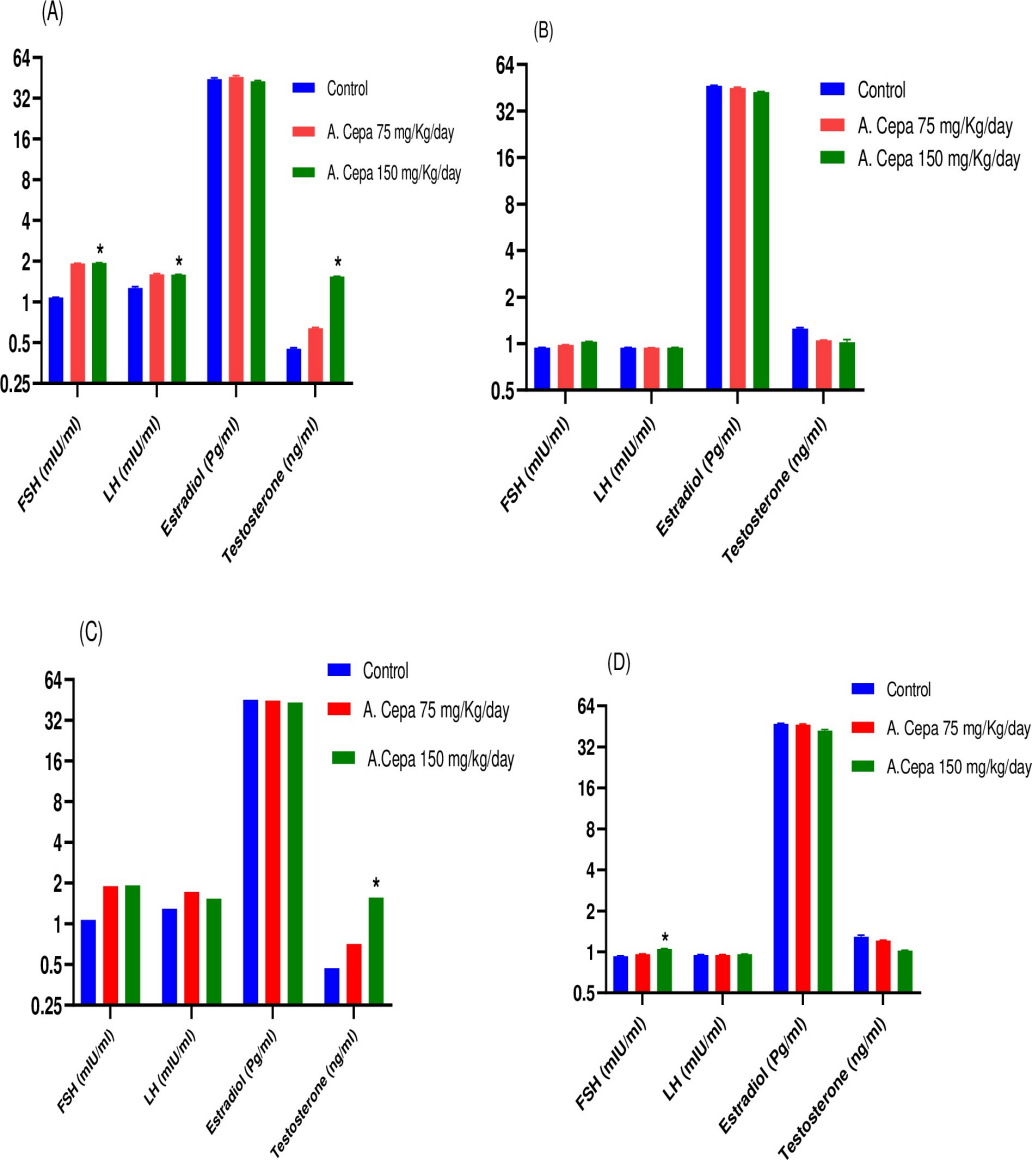
Effect of A. Cepa extract on the hormonal parameters. (A) F_0_ Male (B) F_0_ Female (C) F_1_ Male (D) F_1_ Female. n = 6, Mean ± SEM; *P < 0.05 significant; **F < 0.01 highly significant as compared to control.

FSH, LH, Estradiol and Testosterone levels were tested in the plasma of both control and treated male and female rats. In higher doses, statistically significant raised values of FSH, LH and testosterone were found in F_0_ generation male rats (df 2,15; F 1610; P < 0.05) (df 2,15; F 50.724; P < 0.05) (df 2,15; F 2138; P < 0.05) respectively, when compared to the control. Similarly, F_1_ generation rats also exhibited a significantly raised Testosterone effect (df 2,15; F 3193; P < 0.05). The insignificant result in estradiol was found in male rats (df 2,15; F 3.26, 3.39; P > 0.05) as well as in female rats (df 2,15; F 5.29, 19.26; P > 0.05) respectively, of both F_0_ and F_1_ generation, as shown in Fig 4. In F_1_ generation females, statistically significant raised effect in FSH (df 2,15; F 70.05; P < 0.05) was found.

However, in F_0_ generation female rats, insignificant changes were seen in FSH (df 2,15; F 16.025; P >0.05), LH (df 2,15; F 0.20; P > 0.05) and testosterone (df 2,15; F 16.05; P > 0.05) when compared to control animals.

#### Oxidative parameters

**Fig 5:**
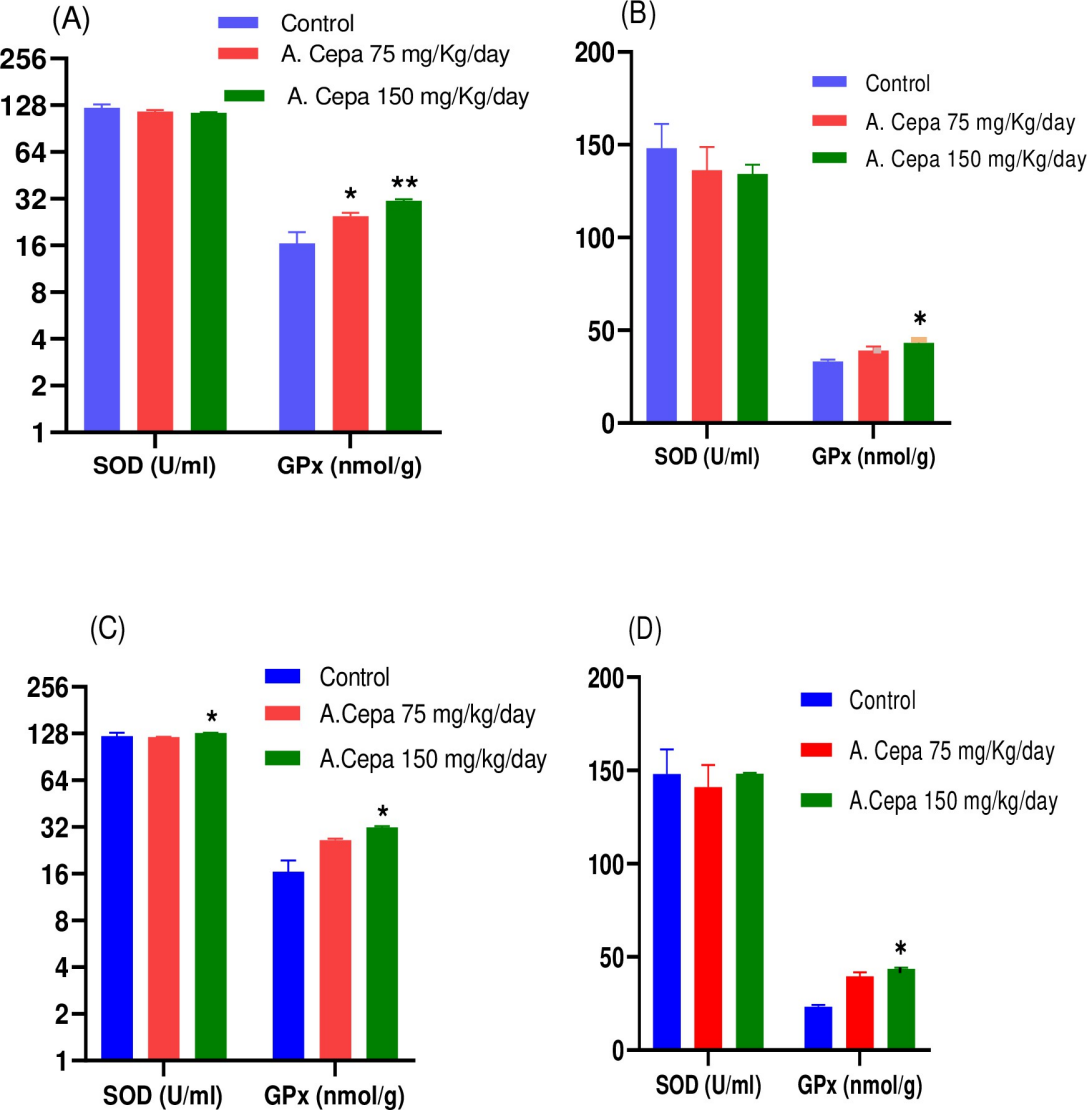
Effect of A. Cepa extract on the oxidative parameters. (A) F_0_ Male (B) F_0_ Female (C) F_1_ Male (D) F_1_ Female. n = 6, Mean ± SEN1; *P < 0.05 significant; ** P < 0.01 highly significant as compared to control.

Glutathione (GPx) levels in treated rats were found to be statistically significant in both F_0_ and F_1_ generations male rats (df 2,15: F 14.20; P < 0.01); (df 2,15: F 19.01; P < 0.05) and female rats (df 2,15; F 14.73, 63.65; P < 0.05) in comparison to control animals. In contrast, insignificant results of SOD were found in F_0_ male rats (df 2,15; F 1.13; P > 0.05) and F_0_ and F_1_ female rats (df 2,15; F 0.461, 0.124; P > 0.05) while F_1_ generation male rats showed a significant raised effect on SOD (df 2,15; F 1.08; P < 0.05) in comparison to the control group as shown in Fig 5.

#### Histopathological evaluation of ovaries

Control ovaries of F_0_ and F_1_ generation rats showed the external cortex surrounded with intact capsule having profuse stroma. A lot of blood vessels parted by unattached connective tissues in the medulla were also observed. The histological ovarian architecture of F_0_ and F_1_ generation treated with low-dose *A*. *Cepa*, animals was similar to controls. However, some primary follicles surrounded beneath the tunica albuginea of an intact primary oocyte can be seen in low-dose treated ovaries, and degenerative changes were also detected in follicles.

**Fig 6:**
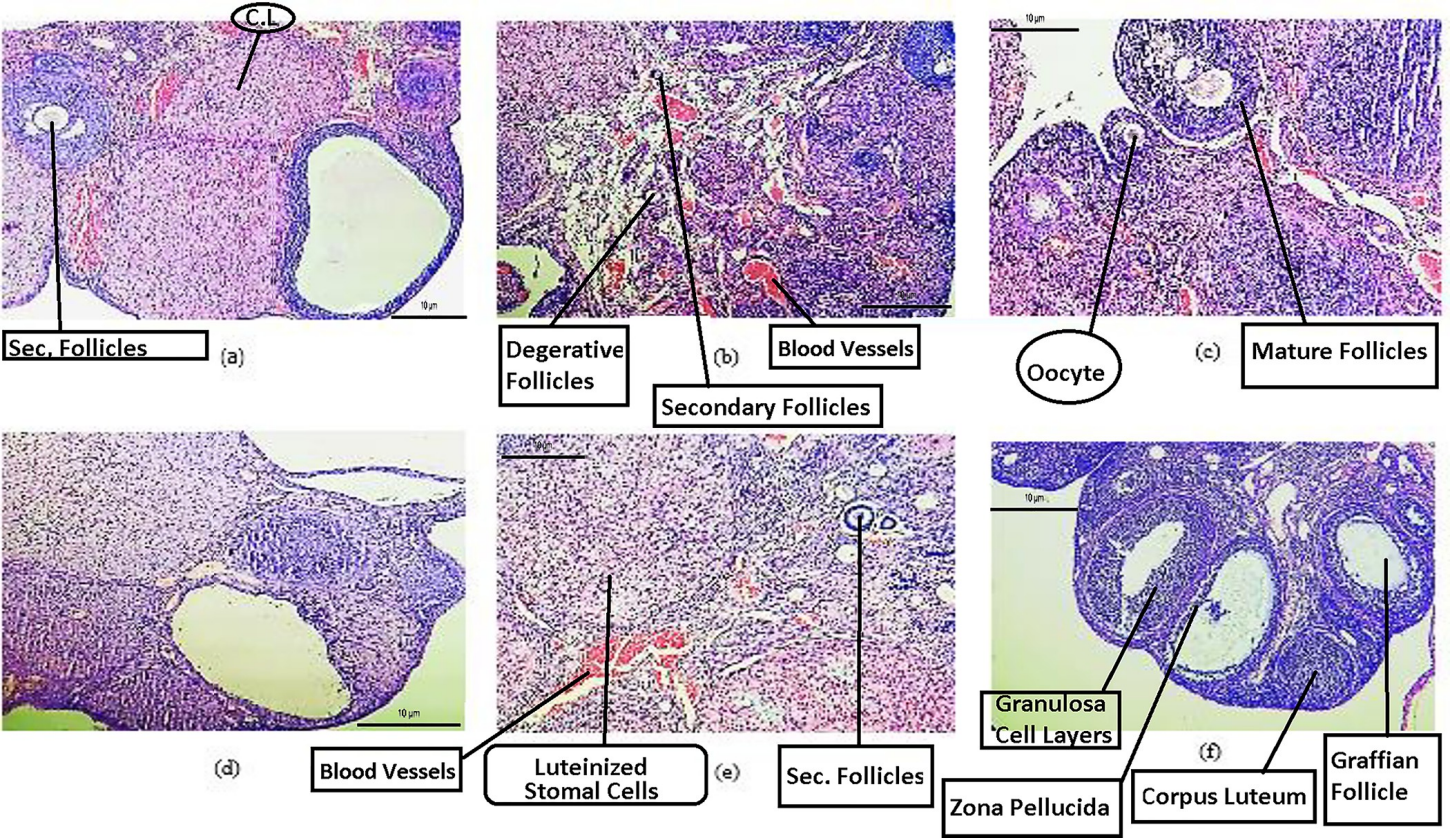
(a) Micrograph, rat ovary F_0_ generation control (10x) (b) Micrograph, rat ovary F_0_ generation low dose *A*. *Cepa* treated (20x) (c) Micrograph, rat ovary F_0_ generation, high dose *A*. *Cepa* treated (20x) (d) Micrograph, rat ovary F_1_ generation control (10x) (e) Micrograph, rat ovary F_1_ generation, *A*. *Cepa* low dose treated (40x) (f) Micrograph, rat ovary F_1_ generation, *A*. *Cepa* high dose treated (10x).

F_0_ and F_1_ generation’s animals of high-dose onion treatment showed Graafian follicle and zona pellucida along with the Nucleus of oocytes. Preantral, small antral, and large antral follicles and corpus luteum of different stages were observed. The number of follicle counts was significantly raised in the F_1_ high-dose treated animals as compared to other groups. Folliculogenesis was seen in all samples as shown in Fig 6.

#### Histopathological evaluation of testes

Microscopic examination of control group testes of F_0_ and F_1_ both generation figures showed an architecture of tubular seminiferous epithelia present normally with occasional Sertoli cells and spermatogenic cells at the interstitial area and base of the tubule having negligible connective tissues at the tubular surroundings.

**Fig 7:**
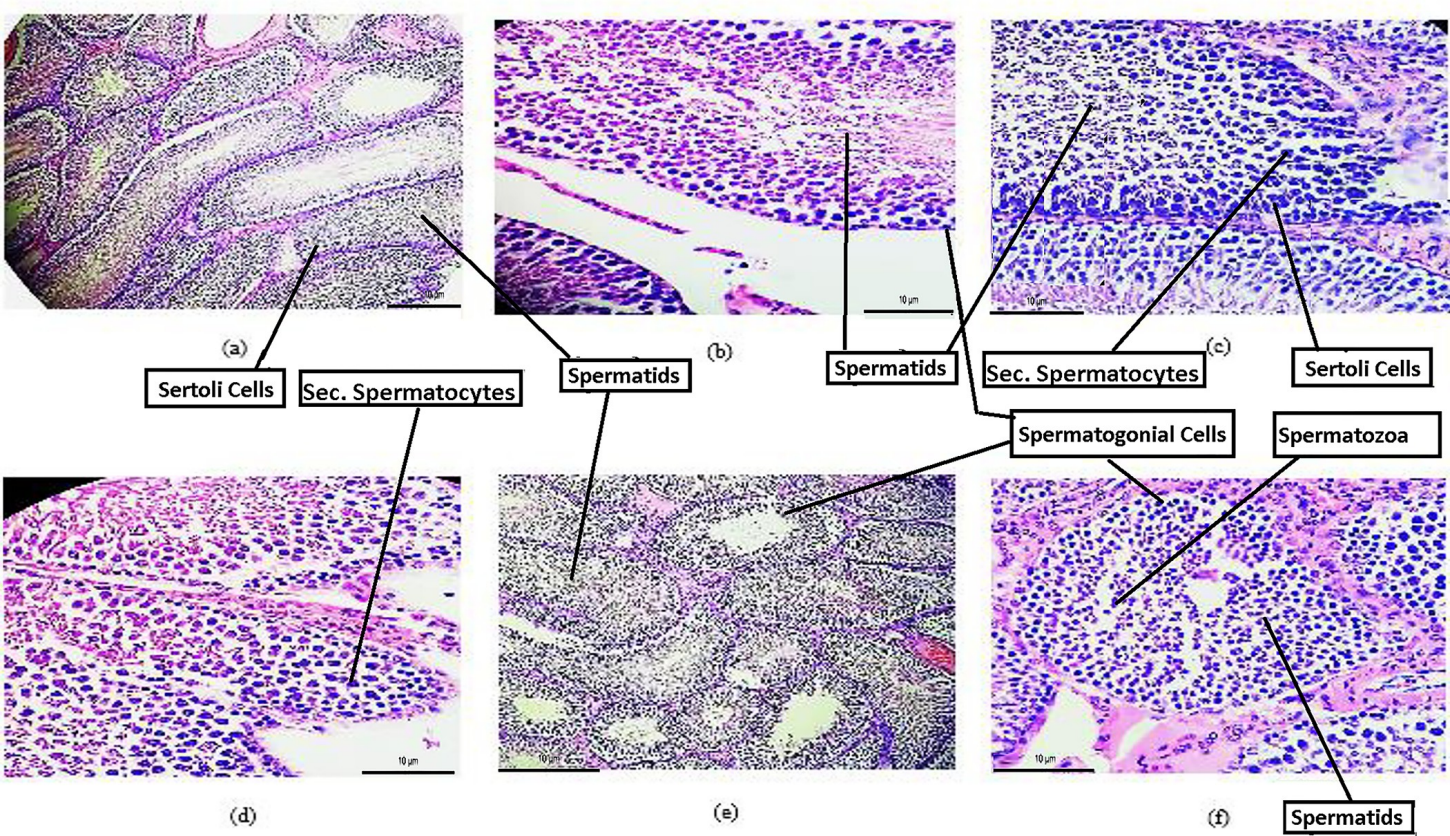
(a) Micrograph, rat testis of F_0_ generation control (20x) (b) Micrograph, rat testis F_0_ generation low dose *A*. *Cepa* treated (40x) (c) Micrograph, rat testis F_0_ generation, high dose *A*. *Cepa* treated (40x) (d) Micrograph, rat testis F_1_ generation control (40x) (e) Micrograph, rat testis F_1_ generation, *A*. *Cepa* low dose treated (20x) (f) Micrograph, rat testis F_1_ generation, *A*. *Cepa* high dose treated (40x).

Microscopic examination of low-dose treated testes of F_0_ and F_1_ generation showed the normal seminiferous tubules having the normal arrangement of Sertoli and spermatogonial cells resting over intact basement membrane. Similarly, in F_0_ and F_1_ generation’s testes, high dose *A*. *Cepa* extract showed normal architecture of seminiferous epithelia of tubules along with Sertoli cells and spermatogenic cells having minimal connective tissues at the tubular surrounding. However, nearer to the tubule’s central part, primary and secondary spermatids at the center of the seminiferous tubule lumen were observed. Increased production of spermatids and spermatozoa was found as compared to the control and low-dose *A*. *Cepa* group as shown in Fig 7.

## Discussion

The therapeutic benefits of *A*. *Cepa* have been acknowledged since ancient civilizations. Important benefits under investigation nowadays include its effect on fecundity and reproduction [23]. Countless experimental researches were conducted to explore the reproductive potential of different herbal extracts to overcome infertility and related problems [7, 51]. *A*. *Cepa* is one of the herbs that have not been investigated widely for its fertility potential, only a few articles are being reported [26, 56]. This two-generation study was established to evaluate the role of *A*. *Cepa* extract in fecundity and the reproductive system.

In the current study, ethanolic extract of *A*. *Cepa* exhibited the presence of different phytochemicals, including tannins, flavonoids, saponins, terpenoids, and polyphenols as well as quantitative data suggest the presence of 63mg/g GAE total polyphenolic content. These findings are in accordance with Prakash et al., 2007 [57], who reported the presence of a rich amount of polyphenols in *A*. *Cepa* extract with promising antioxidant activity. Antioxidant activity of *A*. *Cepa* extract is evaluated by DPPH that showed the highest % inhibition at 100 μg/ml (84.92%). Similarly, in ROS method *A*. *Cepa* extract showed protective efficacy against H_2_O_2_-induced toxicity on neuronal cells. These results suggest dose-dependent radical scavenging activity against DPPH and superoxide anion radicals, which agrees with an earlier study [58].

In screening natural products for pharmacological activity, evaluating the toxic characteristics of medicinal extracts is usually an initial step. During such evaluations, the therapeutic dose determination and mortality rate is the initial step to be conducted [59]. In the Brine shrimp lethality test, the % mortality of *A*. *Cepa* was found at 16.66% at a maximum concentration, which shows that it is safe and significant to use. Similarly, the result of the oral acute toxicity test of *A*. *Cepa* ethanolic extract in-vivo showed that it has a very high safety range when administered orally, even at a maximum dose of 1200 mg/kg. According to Pitchaiah et al. [60], there were no mortality and gross behavioral changes in mice fed with *A*. *Cepa* extract, which suggested its safety in the acute study.

Body weight assessment provides information regarding the health of rodents [32]. The observations showed an insignificant decrease in body weights of F_0_ and F_1_ females following the treatment with lower (T_1_) and higher (T_2_) doses of *A*. *Cepa*. The reduction in body weight may be because *A*. *Cepa* has anti-obesity properties, thus being involved in reducing body weight and food intake in rats [61, 62]. But after week III, the body weights of females of both generations gradually increased during the gestation period [46]. This finding is in accordance with Mirabeau and Samson [63], who reported immune boosting and weight-improving capabilities of *Allium Cepa*.

Our data demonstrated increased pups in both F_1_ and F_2_ generation during the treatment with both doses of onion extract. This indicated that *A*. *Cepa* presented its beneficial actions by augmenting the fertility power of the female rats [26–28]. There was an increase in the litter size of treatment groups in both generations when compared to the control group. Khaki *et al*. [64] also reported increased reproductive performance after administering *A*. *Cepa* juice. In F_1_ off-springs, *A*. *Cepa* treatment caused a slight decrease in the live birth index of the high dose group when compared to control. No effects were observed on mating indices, viability index, and fertility indices on days 4 and 21. However, the live birth index was increased in the F_2_ generation. The outcomes of our study are endorsed further by the investigation of Ara et al. [65], who demonstrated that *A*. *Cepa* extract administration prevented tartrazine-induced noxious infertility in male and female mice. That could be due to polyphenolic compounds present in *A*. *Cepa*, especially quercetin that can repair oxidative damage and hormonal irregularities [66].

Furthermore, in the current study, we have found increased sperm count, volume, viability, and sperm motility by administration of *A*. *Cepa*. This improved result could be due to the antioxidant potential of *A*. *Cepa*, that is a rich source of endogenous and exogenous antioxidants like isorhamnetin and glutathione. It also holds trace minerals and different vitamins; A, B, and C that can assist in preventing damage by reactive oxygen species and protect the tissue from oxidative damage. All these effects may lead to enhanced fertility in males [65].

In our bi-generational experiment (F_0_, F_1_), blood parameters were normal besides platelet count, which increased in both treatment groups. Therefore, *A*. *Cepa* demonstrated potential benefits in maintaining the animal’s good health [67]. A study also reported that onion extract has blood lipid levels reducing potential [68]. Thus, we can conclude that *Allium Cepa* can efficiently decrease the health risk of cardiovascular events in both genders as it is rich in fiber and flavonoids [69, 70].

Reproductive hormones are essential in the development and regular functions of the reproductive system. FSH and Testosterone are important for fulfilling reproductive abilities in males [71]. Studies showed that sex hormones like FSH, LH, and testosterone levels are associated with spermatogenesis [64]. LH activates Leydig cells to produce testosterone while FSH encourages Sertoli cells proliferation. Both these hormones affect testosterone levels that is necessary for sustaining the maturing sperm cells. Moreover, FSH and testosterone impacts the release and growth of spermatids. Thus, an appropriate concentration of FSH, LH and Testosterone is desired for spermatogenesis [72].

FSH, LH, testosterone, and Estradiol levels were estimated in both F_0_ and F_1_ generation animals (male and female rats). In male rats, significantly increased effects were observed by *A*. *Cepa* (150 mg/Kg) on FSH, LH, and Testosterone levels in both generations, depicting its potential as a fertility-enhancing agent. This result is in accordance with the study of Banihani, who reported that *A*. *Cepa* extract consumption enhances testosterone production in males [73]. Testosterone is vital for the maturation and maintenance of the structure and function of the male accessory sex glands. Moreover, insufficiency of this hormone hinders spermatogenic function [74]. Further, Banihani *et al*. suggested the mechanisms by which *A*. *Cepa* enhances testosterone production in males are mainly by enhancing the antioxidant defense mechanism (e.g., antioxidant enzymes, glutathione) in the testis, ameliorating insulin resistance and promoting nitric oxide production in Leydig cells [73].

Increased effects were observed on FSH levels in female rats (F_1_ generation), while in F_0_ generation, FSH levels were insignificant. Additionally, estradiol levels were insignificant and maintained as in the control group. Moreover, LH levels were maintained normal, indicating LH potentiates follicle-stimulating hormone (FSH), stimulates follicle growth, and improves female fertility [75]. Since FSH/LH surge is required for ovulation that is triggered by LH releasing hormone, dependent on estradiol levels [76]. Our results provide the rationale that *A*. *Cepa* in higher doses significantly improved FSH levels by balancing the estradiol and improving fecundity, probably due to the increased pituitary sensitivity to gonadotropin-releasing hormone [77]. These findings are in accordance with Mansouri *et al*., who explained that *A*. *Cepa* extract significantly improves sex hormone production and fertility in electromagnetic field-exposed mice [78]. The changing patterns of sex hormones in the present study are in line with the findings of earlier studies, as Adelakun *et al*. [79], reported that natural extracts have flavonoid contents that decrease oxidative stress and have pro-fertility properties, which are beneficial for fertility.

Recent studies have reported the anti-oxidant properties of *A*. *Cepa* are due to flavonoid quercetin [80]. Quercetin-induced upregulation of glutathione levels may elucidate the antioxidant effects of *Allium Cepa* [81]. *A*. *Cepa* extract increased the level of antioxidants in the body, such as glutathione [82]. Our study also confirmed the anti-oxidant potential of *A*. *Cepa* extract by increasing the glutathione (GPx) levels of both male and female Wistar rats. Present findings for glutathione (GPx) depicted the highest correlation between antioxidant potential (DPPH, ROS) and total phenolic contents in *A*. *Cepa* extract.

In Histopathological examination, female rats exhibited an improved number of follicles count as well as Folliculogenesis in the F_1_ high-dose treated animals. In addition, the male testis showed improved architecture of seminiferous tubular epithelia along with Sertoli cells and increased spermatogenic cells. These all above results are in line of agreement with Ara *et al*. [65], who reported that onion extract significantly ameliorated tartrazine-induced histopathological changes with improved architecture, granulosa cells, and corpus luteum. This could be due to the antioxidant activity of *A*. *Cepa* extract against reproductive deteriorations.

## Conclusion

In conclusion, this study revealed that administration of *A*. *Cepa* ethanolic extract for seven months had beneficial effects on both male and female fecundity processes by enhancing ovulation and spermatogenesis with no toxic effect in both sexes. This could be related to its lipid-lowering action as manifested by the modulation of serum total cholesterol as well as its antioxidant and androgenic effects. Therefore, it is suggested that daily consumption of *A*. *Cepa* may provide an effective strategy to improve fertility. However, further infertility induced model studies are needed to investigate the effects of its active constituents and pure compounds in clinical trials.

## Supporting information

S1 File (ZIP)
