# Supplementary material for: The beneficial effect of Allium Cepa bulb extract on reproduction of rats; A two-generation study on fecundity and sex hormones
Source: PLoS One. 2024 Mar 14;19(3):e0294999. doi: 10.1371/journal.pone.0294999 (PMC10939208; doi:10.1371/journal.pone.0294999)
Supplement: S1 File — (ZIP) [file pone.0294999.s001.zip › A Cepa - lipid parameters F1.docx]

**Effect of A. Cepa extract on the biochemical parameters in both genders of F_1_ generation rats as compared to control.**

| MALE | | | | FEMALE | | | | | |
| --- | --- | --- | --- | --- | --- | --- | --- | --- | --- |
|  | Control | T1 | T2 |  | Control | T1 | T2 |  |  |

| Cholesterol (mg/dl) | 161.8 ± 5.65 | 160.66 ± 2.41 | 138 ± 5.69^**^ |  | 139.8 ± 0.94 | 135.16 ± 0.70 | 128.16 ± 0.90 ^*^ |
| --- | --- | --- | --- | --- | --- | --- | --- |
| Triglycerides (mg/dl) | **130.3± 1.97** | **122.83 ± 6.32** | **117.6 ± 2.13 ^*^** |  | **121.16 ± 1.32** | **109.33 ± 1.85** | **98.66 ± 1.52^*^** |
| HDL (mg/dl) | **34.33 ± 1.85** | **35 ± 1.59** | **40.83 ± 1.04 ^*^** |  | **41.8 ± 0.74** | **44.83 ± 0.70** | **46.66 ±1.62 ^*^** |
| LDL (mg/dl) | **105.66± 7.78** | **89 ± 5.18** | **67.33± 1.11 ^**^** |  | **76.33 ± 0.42** | **72.33 ± 0.42** | **65.16 ± 0.40 ^*^** |
| VLDL (mg/dl) | **26.83 ± 0.98** | **25 ± 2.06** | **24.66 ± 1.14*** |  | **22.83 ± 0.83** | **19.83 ± 1.13** | **17.16 ± 0.47 ^*^** |

**F_0_ presents Parent Generation while F_1_ presents, 1^st^ Generation, T_1_ shows low dose group while T_2_ shows high dose group.**

**n = 6, Mean ± SEM; *P < 0.05 significant; ** P < 0.01 highly significant as compared to control.**
