# Supplementary material for: The beneficial effect of Allium Cepa bulb extract on reproduction of rats; A two-generation study on fecundity and sex hormones
Source: PLoS One. 2024 Mar 14;19(3):e0294999. doi: 10.1371/journal.pone.0294999 (PMC10939208; doi:10.1371/journal.pone.0294999)
Supplement: S1 File — (ZIP) [file pone.0294999.s001.zip › Biochemical Lipid Parameters F0 Generation.docx]

**Effect of A. Cepa extract on the biochemical parameters in both genders of F_0_ generation rats as compared to control.**

| MALE | | | | FEMALE | | | | | |
| --- | --- | --- | --- | --- | --- | --- | --- | --- | --- |
|  | **Control** | **T1** | **T2** |  | **Control** | **T1** | **T2** |  |  |

| Cholesterol (mg/dl) | 162.5 ± 5.71 | 152.16 ± 5.43 | 135 ± 6.08^**^ |  | 136.50 ± 0.99 | 133.33 ± 1.45 | 129.33 ± 1.33^*^ |
| --- | --- | --- | --- | --- | --- | --- | --- |
| Triglycerides (mg/dl) | 129.66± 2.29 | 114.33 ± 8.67 | 108 ± 3.13 **^*^** |  | 101.16 ± 1.16 | 100.83 ± 1.01 | 93.66 ± 1.08**^**^** |
| HDL (mg/dl) | 33.83 ± 2.13 | 34.50 ± 1.33 | 40 ± 0.85 **^*^** |  | 42 ± 0.73 | 46.5 ± 0.56 **^*^** | 47.66 ±1.83 **^**^** |
| LDL (mg/dl) | 104.33± 7.50 | 87.33 ± 5.42 | 66 ± 0.96 **^**^** |  | 66.16 ± 1.19 | 62.33 ± 0.42 **^*^** | 60.33 ± 0.49 **^**^** |
| VLDL (mg/dl) | 25.16 ± 0.87 | 24.33 ± 2.07 | 26 ± 1.29 |  | 20 ± 1.06 | 19.33 ± 1.14 | 16.5 ± 0.42 **^*^** |

**F_0_ presents Parent Generation while F_1_ presents, 1^st^ Generation, T_1_ shows low dose group while T_2_ shows high dose group.**

**n = 6, Mean ± SEM; *P < 0.05 significant; ** P < 0.01 highly significant as compared to control.**
