# Supplementary material for: The beneficial effect of Allium Cepa bulb extract on reproduction of rats; A two-generation study on fecundity and sex hormones
Source: PLoS One. 2024 Mar 14;19(3):e0294999. doi: 10.1371/journal.pone.0294999 (PMC10939208; doi:10.1371/journal.pone.0294999)
Supplement: S1 File — (ZIP) [file pone.0294999.s001.zip › Hematological Parameters F0.docx]

**Effect of A. Cepa extract on the hematological parameters in both genders of F0 generation rats as compared to control.**

| MALE | | | | FEMALE | | | | | |
| --- | --- | --- | --- | --- | --- | --- | --- | --- | --- |
|  | Control | T1 | T2 |  | Control | T1 | T2 |  |  |
| Hemoglobin (g/dl) | 12.71 ± 0.37 | 11.33±0.19 | 11.93 ± 0.09 |  | 14.83 ± 0.15 | 14.83 ± 0.12 | 14.21 ± 0.19 **^*^** |  |  |
| Haemtocrit (%) | 42.58 ± 1.11 | 39.01 ± 1.46 | 36.40 ± 0.17 |  | 45.21 ± 0.19 | 45.28 ± 0.16 | 44.86 ± 0.20 |  |  |
| RBCs(× 10^12/L) | 5.00 ± 0.02 | 4.66 ± 0.15 | 4.70 ± 0.69 |  | 4.41 ± 0.01 | 4.42 ± 0.016 | 44.41 ± 0.005 |  |  |
| WBCs(× 10^9/L) | 4.25 ± 0.37 | 3.83 ± 0.17 | 3.93 ± 0.07 |  | 6.35 ± 0.07 | 6.16 ± 0.14 | 66.01 ± 0.18 |  |  |
| Platelets(×10^9/L) | 678 ± 53.19 | 858 ± 29.55 **^*^** | 948±14.23 **^**^** |  | 340.33 ± 1.20 | 341.83 ± 1.30 | 359 ±16.72 |  |  |
