# Supplementary material for: The beneficial effect of Allium Cepa bulb extract on reproduction of rats; A two-generation study on fecundity and sex hormones
Source: PLoS One. 2024 Mar 14;19(3):e0294999. doi: 10.1371/journal.pone.0294999 (PMC10939208; doi:10.1371/journal.pone.0294999)
Supplement: S1 File — (ZIP) [file pone.0294999.s001.zip › Hematological Parameters F1.docx]

**Effect of A. Cepa extract on the hematological parameters in both genders of F1 generation rats as compared to control.**

| MALE | | | | FEMALE | | | | | |
| --- | --- | --- | --- | --- | --- | --- | --- | --- | --- |
|  | Control | T1 | T2 |  | Control | T1 | T2 |  |  |
| Hemoglobin (g/dl) | 12.78 ± 0.34 | 12.65 ±0.15 | 12.78 ± 0.22 |  | 12.61 ± 0.04 | 12.36 ± 0.07 | 12.79 ± 0.06 |  |  |
| Haemtocrit (%) | 43.06 ± 1.05 | 42.26 ± 0.47 | 42.50 ± 0.70 |  | 42.10 ± 0.24 | 43.36 ± 0.09 | 43.41 ± 0.13 |  |  |
| RBCs (× 10^12/L) | 4.95 ± 0.02 | 5.01 ± 0.12 | 5.30 ± 0.03 |  | 4.55 ± 0.06 | 4.82 ± 0.01 | 45.22 ± 0.01 |  |  |
| WBCs (× 10^9/L) | 4.69 ± 0.30 | 5.48 ± 0.18 | 4.60 ± 0.14 |  | 4.55 ± 0.06 | 5.13 ± 0.09 | 64.93 ± 0.009 |  |  |
| Platelets(×10^9/L) | 678 ± 53.19 | 585.1 ± 1.57 | 520.1 ± 1.04 **^*^** |  | 570 ± 3.43 | 594.1 ± 1.16 | 551 ± 0.36^*^ |  |  |
