# Supplementary material for: The beneficial effect of Allium Cepa bulb extract on reproduction of rats; A two-generation study on fecundity and sex hormones
Source: PLoS One. 2024 Mar 14;19(3):e0294999. doi: 10.1371/journal.pone.0294999 (PMC10939208; doi:10.1371/journal.pone.0294999)
Supplement: S1 File — (ZIP) [file pone.0294999.s001.zip › Hormonal parameters F0 generation.docx]

**Effect of A. Cepa extract on the Hormonal parameters of F0 generation rats as compared to control.**

| MALE | | | | | FEMALE | | | | | | | |
| --- | --- | --- | --- | --- | --- | --- | --- | --- | --- | --- | --- | --- |
| Parameters | **Control** | **T1** | **T2** |  | **Control** | **T1** | | **T2** | | |  | |
| Hormones |  |  |  |  |  |  | |  | | |  | |
| FSH (mIU/ml) | 1.08± 0.004 | 1.92 ± 0.01 | 1.94± 0.01^*^ |  | 0.94 ± 0.01 | | 0.98 ± 0.01 | | 1.03 ± 0.01 | | |  |
| LH (mIU/ml) | 1.27 ± 0.03 | 1.60 ± 0.02 | 1.59 ±0.01**^*^** |  | 0.94± 0.009 | | 0.94± 0.008 | | | 0.94 ± 0.01 | |  |
| Estradiol (Pg/ml) | 44.33± 0.98 | 46 ± 1.06 | 42.66± 0.66 |  | 46.66± 0.76 | | 45.16± 1.16 | | | 42.50± 0.76 | |  |
| Testosterone (ng/ml) | 0.45 ± 0.01 | 0.64 ± 0.01 | 1.54 ±0.01**^*^** |  | 1.25 ± 0.05 | | 1.05 ± 0.01 | | | 1.02 ± 0.10 | |  |

**F_0_ presents Parent Generation, while F_1_ presents 1^st^ Generation, T_1_ shows low dose group while T_2_ shows high dose group.**

**n = 6. Mean ± SEM; *P < 0.05 significant; ** P < 0.01 highly significant as compared to control.**
