# Supplementary material for: The beneficial effect of Allium Cepa bulb extract on reproduction of rats; A two-generation study on fecundity and sex hormones
Source: PLoS One. 2024 Mar 14;19(3):e0294999. doi: 10.1371/journal.pone.0294999 (PMC10939208; doi:10.1371/journal.pone.0294999)
Supplement: S1 File — (ZIP) [file pone.0294999.s001.zip › Hormonal parameters F1 generation.docx]

**Effect of A. Cepa extract on the Hormonal parameters of F1 generation rats as compared to control.**

| MALE | | | | | FEMALE | | | | | | | |
| --- | --- | --- | --- | --- | --- | --- | --- | --- | --- | --- | --- | --- |
| Parameters | **Control** | **T1** | **T2** |  | **Control** | **T1** | | **T2** | | |  | |
| Hormones |  |  |  |  |  |  | |  | | |  | |
| FSH (mIU/ml) | 1.07± 0.003 | 1.90 ± 0.01 | 1.92± 0.009 |  | 0.93 ± 0.006 | | 0.96± 0.007 | | 1.05 ± 0.007^*^ | | |  |
| LH (mIU/ml) | 1.29 ± 0.03 | 1.72 ± 0.007 | 1.53 ±0.01 |  | 0.95± 0.005 | | 0.95± 0.005 | | | 0.96 ± 0.005 | |  |
| Estradiol (Pg/ml) | 45.33± 0.71 | 44.83 ± 0.54 | 43.31± 0.41 |  | 47.16± 0.54 | | 46.56± 0.55 | | | 42.16 ± 0.74 | |  |
| Testosterone (ng/ml) | 0.47±0.005 | 0.71 ± 0.002 | 1.56 ±0.01**^*^** |  | 1.29 ± 0.04 | | 1.21 ± 0.01 | | | 1.02 ± 0.01 | |  |

**F_0_ presents Parent Generation, while F_1_ presents 1^st^ Generation, T_1_ shows low dose group while T_2_ shows high dose group.**

**n = 6. Mean ± SEM; *P < 0.05 significant; ** P < 0.01 highly significant as compared to control.**
