# Supplementary material for: The beneficial effect of Allium Cepa bulb extract on reproduction of rats; A two-generation study on fecundity and sex hormones
Source: PLoS One. 2024 Mar 14;19(3):e0294999. doi: 10.1371/journal.pone.0294999 (PMC10939208; doi:10.1371/journal.pone.0294999)
Supplement: S1 File — (ZIP) [file pone.0294999.s001.zip › Oxidative Parameters F0.docx]

**Effect of A. Cepa extract on the oxidative parameters of F0 generation as compared to control.**

| MALE | | | | | FEMALE | | | |
| --- | --- | --- | --- | --- | --- | --- | --- | --- |
|  | Control | T1 | T2 |  | Control | T1 | T2 |  |
| SOD (U/ml) | 122.83±6.79 | 116.66±2.26 | 114.16±1.07 |  | 148 ± 13.25 | 136.33±12.39 | 134.3 ± 4.99 |  |
| Glutathione  (GPx)  (nmol/g) | 16.50± 2.98 | 24.66±1.28 **^*^** | 31 ± 0.77 **^**^** |  | 33.33 ± 0.84 | 39.16 ±2.07 | 43.83 ± 0.79 **^*^** |  |
|  |  |  |  |  |  |  |  |  |

**F_0_ presents Parent Generation, while F_1_ presents 1^st^ Generation, T_1_ shows low dose group while T_2_ shows high dose group.**

**n = 6. Mean ± SEM; *P < 0.05 significant; ** P < 0.01 highly significant as compared to control.**
