# Supplementary material for: The beneficial effect of Allium Cepa bulb extract on reproduction of rats; A two-generation study on fecundity and sex hormones
Source: PLoS One. 2024 Mar 14;19(3):e0294999. doi: 10.1371/journal.pone.0294999 (PMC10939208; doi:10.1371/journal.pone.0294999)
Supplement: S1 File — (ZIP) [file pone.0294999.s001.zip › Oxidative Parameters F1.docx]

**Effect of A. Cepa extract on the oxidative parameters of F1 generation as compared to control.**

| MALE | | | | | FEMALE | | | |
| --- | --- | --- | --- | --- | --- | --- | --- | --- |
|  | Control | T1 | T2 |  | Control | T1 | T2 |  |
| SOD (U/ml) | 122.8±6.79 | 121.5±0.42 | 129.1 ±0.30^*^ |  | 148 ± 13.25 | 141 ±11.87 | 148.2 ± 0.54 |  |
| Glutathione (GPx)  (nmol/g) | 16.50± 2.98 | 26.33±0.61 | 31.83± 0.47**^*^** |  | 23.33 ± 0.84 | 39.66 ±2.02 | 43.5 ± 0.76**^*^** |  |
|  |  |  |  |  |  |  |  |  |

**F_0_ presents Parent Generation, while F_1_ presents 1^st^ Generation, T_1_ shows low dose group while T_2_ shows high dose group.**

**n = 6. Mean ± SEM; *P < 0.05 significant; ** P < 0.01 highly significant as compared to control.**
