# Supplementary figures and images for: The beneficial effect of Allium Cepa bulb extract on reproduction of rats; A two-generation study on fecundity and sex hormones
Source: PLoS One. 2024 Mar 14;19(3):e0294999. doi: 10.1371/journal.pone.0294999 (PMC10939208; doi:10.1371/journal.pone.0294999)

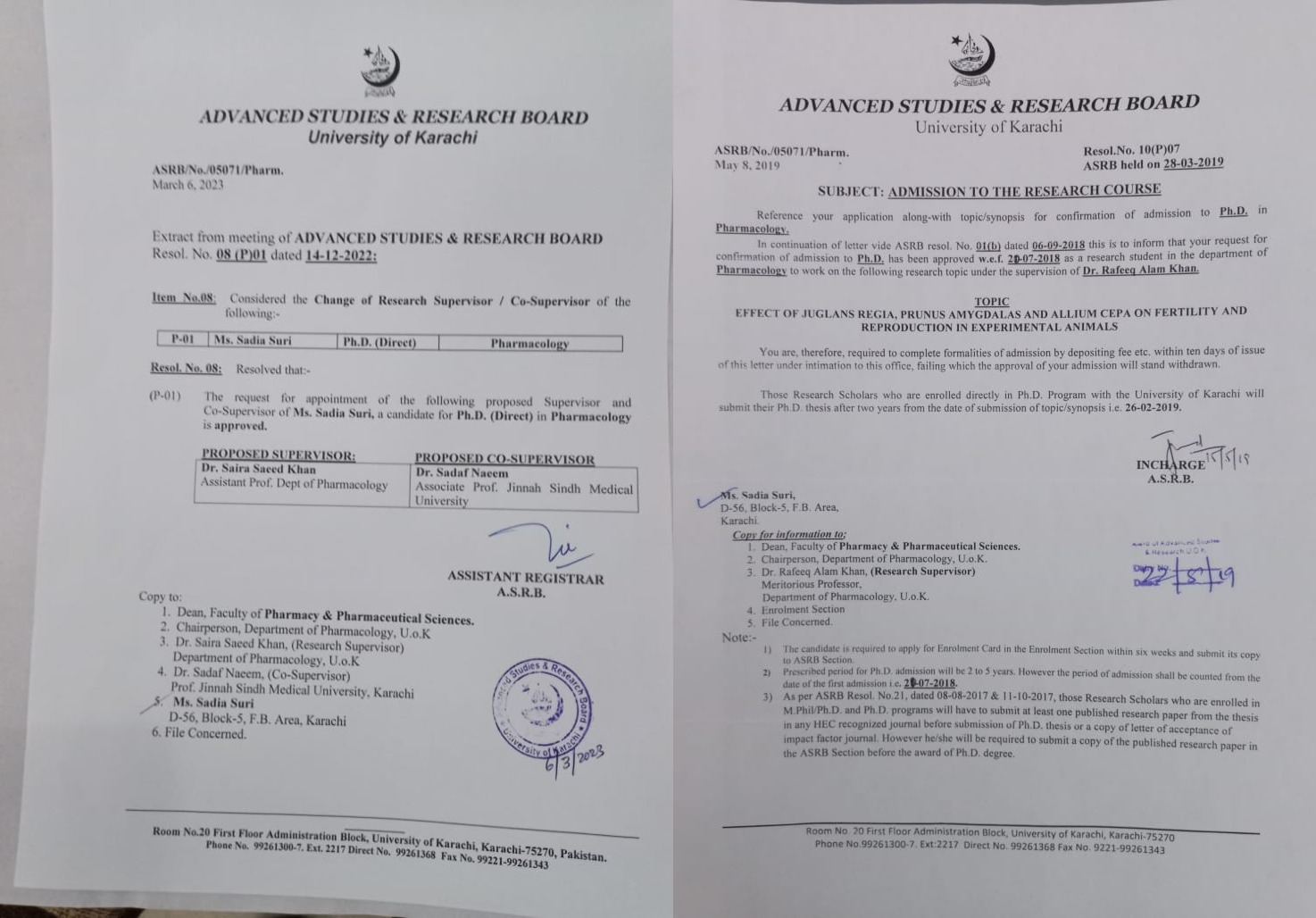

Supplement: S1 File — (ZIP) [file pone.0294999.s001.zip › ethichal approval.jpg]
